# Systemic infection modifies the neuroinflammatory response in late stage Alzheimer’s disease

**DOI:** 10.1186/s40478-018-0592-3

**Published:** 2018-09-07

**Authors:** Sonja Rakic, Yat M. A. Hung, Matthew Smith, Denise So, Hannah M. Tayler, William Varney, Joe Wild, Scott Harris, Clive Holmes, Seth Love, William Stewart, James A. R. Nicoll, Delphine Boche

**Affiliations:** 1 Clinical Neurosciences, Clinical and Experimental Sciences Academic Unit, Faculty of Medicine, University of Southampton, Southampton, UK; 2 Dementia Research Group, University of Bristol Medical School, Learning & Research, Southmead Hospital, Bristol, UK; 3 Public Health Sciences and Medical Statistics, Faculty of Medicine, University of Southampton, Southampton, UK; 4 Memory Assessment and Research Centre, Moorgreen Hospital, Southampton, UK; 5 Department of Neuropathology, Queen Elizabeth University Hospital, Glasgow, UK; 6 Institute of Neuroscience and Psychology, University of Glasgow, Glasgow, UK; 7 Department of Cellular Pathology, University Hospital Southampton NHS Foundation Trust, Southampton, UK

**Keywords:** Alzheimer’s disease, Systemic infection, Neuroinflammation, Human brain, Microglia

## Abstract

Clinical studies indicate that systemic infections accelerate cognitive decline in Alzheimer’s disease. Animal models suggest that this may be due to enhanced pro-inflammatory changes in the brain. We have performed a *post-mortem* human study to determine whether systemic infection modifies the neuropathology and in particular, neuroinflammation, in the late-stage of the disease.

Sections of cerebral cortex and underlying white matter from controls and Alzheimer's patients who died with or without a terminal systemic infection were immunolabelled and quantified for: (i) Αβ and phosphorylated-tau; (ii) the inflammation-related proteins Iba1, CD68, HLA-DR, FcγRs (CD64, CD32a, CD32b, CD16), CHIL3L1, IL4R and CCR2; and (iii) T-cell marker CD3. In Alzheimer's disease, the synaptic proteins synaptophysin and PSD-95 were quantified by ELISA, and the inflammatory proteins and mRNAs by MesoScale Discovery Multiplex Assays and qPCR, respectively.

Systemic infection in Alzheimer's disease was associated with decreased CD16 (*p* = 0.027, grey matter) and CD68 (*p* = 0.015, white matter); increased CD64 (*p* = 0.017, white matter) as well as increased protein expression of IL6 (*p* = 0.047) and decreased IL5 (*p* = 0.007), IL7 (*p* = 0.002), IL12/IL23p40 (*p* = 0.001), IL15 (*p* = 0.008), IL16 (*p* < 0.001) and IL17A (*p* < 0.001). Increased expression of anti-inflammatory genes CHI3L1 (*p* = 0.012) and IL4R (*p* = 0.004) were detected in this group. T-cell recruitment to the brain was reduced when systemic infection was present. However, exposure to systemic infection did not modify the pathology. In Alzheimer's disease, CD68 (*p* = 0.026), CD64 (*p* = 0.002), CHI3L1 (*p* = 0.016), IL4R (*p* = 0.005) and CCR2 (*p* = 0.010) were increased independently of systemic infection.

Our findings suggest that systemic infections modify neuroinflammatory processes in Alzheimer's disease. However, rather than promoting pro-inflammatory changes, as observed in experimental models, they seem to promote an anti-inflammatory, potentially immunosuppressive, environment in the human brain.

**Electronic supplementary material:**

The online version of this article (10.1186/s40478-018-0592-3) contains supplementary material, which is available to authorized users.

## Introduction

Systemic infections lead to the development of “sickness behaviour”, clinical features of which include fever, depression, apathy, self-reported ill health and attentional deficits [18]. At least in animal models this is, in part, mediated by the transient production of pro-inflammatory cytokines by microglia, in turn activated by cytokines and other inflammatory mediators generated by peripheral immune cells [17, 18]. In humans, the clinical features of sickness behaviour are usually considered benign and transitory. However, animal studies have shown that, when microglia are “primed” by early neurodegeneration, systemic infection can switch the central innate immune response from a hybrid of pro- and anti-inflammatory phenotypes to a more tissue-damaging environment, with enhanced and prolonged pro-inflammatory cytokine synthesis in the brain, symptoms of sickness behaviour, and increased neuronal death [15, 16].

Microglia are highly plastic and dynamic cells [47] that adapt their behaviour and morphology to adjust to their environment, adopting different profiles and morphologies [8, 25]. It was proposed that in patients with neurodegenerative disease, systemic infection would exacerbate symptoms of the disease, increase tissue injury and accelerate disease progression [54]. In the absence of neurodegenerative disease, *post-mortem* studies have shown that systemic infection is associated with increased activation of vascular endothelial cells, perivascular macrophages [65] and microglia [38, 61]. In prospective clinical studies of people with Alzheimer’s disease (AD), systemic infection with raised peripheral pro-inflammatory cytokines is associated with a marked increase in the rate of cognitive decline and in the neuropsychiatric features of sickness behaviour [29, 30]. This supports the hypothesis that microglia in the diseased brain, in a relatively benign but primed inflammatory state [55], may be activated by signalling molecules from systemic infection [59], to produce cytokines and other molecules that promote neuronal dysfunction and degeneration.

To explore the effects of systemic infection on the human AD brain, we have conducted a *post-mortem* study in which Alzheimer’s cases were selected on the basis of the presence or absence of systemic infection at the time of death. We investigated whether systemic infection modifies the neuroinflammatory environment and thus the microglial profile, and assessed the potential consequences on AD-associated pathologies.

## Materials and methods

### Cases

Autopsy-acquired brain tissue from 108 donors was sourced from the South West Dementia Brain Bank (University of Bristol) and BRAIN UK (Queen Elizabeth University Hospital, Glasgow). Clinical history as included in *post-mortem* reports and information on the death certificate was used to subdivide cases according to whether systemic infection was or was not recorded as cause of death into four subgroups: cognitive and neuropathological controls (Ctrl), who died without systemic infection (Ctrl-, *n* = 24) or with systemic infection (Ctrl+, *n* = 16); and AD patients, who died without systemic infection (AD-, *n* = 28) or with systemic infection (AD+, *n* = 40). Alzheimer’s cases had a clinical diagnosis of AD made during life and satisfied *post-mortem* neuropathological consensus criteria for AD [31] without having any other significant brain pathologies such as stroke, primary or metastatic tumour, or traumatic lesions. The causes of death in the control and AD groups without systemic infection included cardiovascular disease and non-brain tumours. In the control and AD groups with systemic infection, death was attributed in most cases to bronchopneumonia and urinary tract infection. The characteristics of the groups are presented in Table 1.

**Table 1:** Demographic, clinical and post-mortem characteristics of controls and Alzheimer’s cases

Cases	Ctrl- (*n* = 24)	Ctrl+ (*n* = 16)	AD- (*n* = 28)	AD+ (*n* = 40)
Gender	12F:12M	7F:9M	16F:12M	25F:15M
Age of Death (years, mean±SD)	80.4±10.4	82.1±9.5	81.1±6.1	82±7.4
Age of AD onset (years, mean±SD)	n/a	n/a	72.7±7.7	74.3±8.9
Duration of AD (years, mean±SD)	n/a	n/a	8.4±4.3	7.7±4.0
Braak Stage	0-II: 18	0-II: 11	0-II: 0	0-II: 0
	III-IV: 2	III-IV: 2	III-IV: 6	III-IV: 6
	V-VI: 0	V-VI: 0	V-VI: 22	V-VI: 34
Cause of death				
*Cardiovascular disease*	20/24 (83.3%)		7/28 (25%)	
*Non-brain tumour*	2/24 (8.3%)		5/28 (17.9%)	
*Other*	^a^2/24 (8.3%)	^a^2/16 (12.5%)	^b^16/28 (57.1%)	^a^3/40 (7.5%)
*Bronchopneumonia*		12/16 (75%)		32/40 (80%)
*Urinary Tract Infection*		2/16 (12.5%)		5/40 (12.5%)
*APOE* genotype				
*ε4/−*	2/19 (10.5%)	2/10 (20%)	9/23 (39.1%)	15/36 (41.7%)
*ε4/ε4*	1/19 (5.3%)	0/10 (0%)	5/23 (21.7%)	8/36 (22.2%)
*Post-mortem* delay (hours, mean±SD)	34.6±18.5	50.1±27.4	37.8±26.8	48.2±23.3
pH	n/a	n/a	6.1±0.4	6.1±0.3

*Ctrl* neurologically/cognitively normal controls, *AD* Alzheimer’s disease, − died without systemic infection, + died with systemic infection, *F* female, *M* male, *RIN* RNA integrity number, *n/a* not-applicable, *SD* standard deviation

Braak staging and *APOE* genotyping were not available for all cases

other cause of death included: ^a^bowel obstruction, ruptured abdominal aortic aneurysm, fall (fractured femur); ^b^Alzheimer’s disease

The inferior parietal lobe (Brodmann area 40), an area of cerebrum typically affected by AD pathology [45], was investigated in all cases. Formalin-fixed paraffin embedded tissue was used for the immunodetection of neuropathological and neuroinflammatory markers in the control and AD groups. Fresh frozen tissue available only for the AD groups with and without systemic infection and selected on a pH > 6.0 to ensure RNA integrity [4, 56], was used for detection of synaptic proteins by ELISA, and for detection of inflammation-related proteins and mRNA by MesoScale Discovery (MSD) multiplex assays and quantitative (q) PCR.

### Immunohistochemistry

Immunohistochemistry was performed on 4 μm paraffin sections in several separate batches, with each batch containing cases from all groups (Ctrl-, Ctrl+, AD-, AD+) to ensure comparability of immunolabelling. All experiments included a negative control slide incubated in buffer with no primary antibody, and a positive control slide containing a specific tissue type known to express the protein of interest (e.g. tonsil). Details of the primary antibodies including immune functions and pre-treatments are presented in Additional file 1: Table S1. Biotinylated secondary antibodies rabbit anti-goat and swine anti-rabbit were from Dako (Glostrup, Denmark) and goat anti-mouse from Vector Laboratories (Peterborough, UK). Bound antibodies were visualized using the avidin–biotin–peroxidase complex method (Vectastain Elite, Vector Laboratories) with 3,3′-diaminobenzidine as chromogen and 0.05% hydrogen peroxide as substrate (Vector Laboratories). All sections were counterstained with haematoxylin, then dehydrated before mounting in DePeX (VWR International, Lutterwort, UK).

#### Quantification

Quantification was blinded to the case designation and performed separately on the grey matter and white matter in the same sulcus of the inferior parietal lobule for all cases, as determined by an experienced neuropathologist (JARN). For each case, 30 images of grey matter were acquired by the Olympus dotSlide virtual microscopy system under a × 20 objective. The images were obtained in a zigzag sequence to ensure sampling of all six cortical layers as previously published [44, 74]. An additional 30 images were obtained of the subcortical white matter. Quantitative image analysis was carried out using ImageJ (version 1.49u, Wayne Rasband, NIH, USA). For each antibody, a specific threshold was determined to quantify the area fraction of each image labelled by the antibody and expressed as protein load (%), and the mean value was calculated for each case for each antibody.

For T cells, semi-quantitative analysis was performed manually and based on assessment of the whole section under a × 10 objective. CD3+ T-cells were identified as present or absent in the vasculature and parenchyma of the grey and white matter. Subsequent analysis was based on the percentage of cases with T cells present or absent in each subgroup.

### ELISA

ELISA was carried out to quantify the presynaptic protein synaptophysin (SYP), postsynaptic density protein 95 (PSD95), and neuron-specific enolase (NSE) – a neuronal marker used to control for variation in neuronal content between samples. The ratio of synaptophysin to PSD95 was calculated as an indicator of selective pre- or post-synaptic loss. 100 mg of fresh frozen grey matter from AD cases (*n* = 67) was homogenised in lysis buffer at a tissue concentration of 20% *w*/*v* [66] and total protein measured by Pierce Coomassie (Bradford) Protein Assay Kit (Thermo Fisher Scientific, Waltham, USA). Non-specific binding was blocked with blocking buffer (1% BSA-PBS). All measurements were corrected for total protein concentration. SYP and PSD95 values were subsequently adjusted for NSE concentration.

#### SYP and NSE measurements

SYP and NSE were measured by sandwich ELISA and PSD95 by indirect ELISA [52, 62]. The capture antibody, SYP (Abcam, Cambridge, UK) or NSE (Enzo Life Sciences, Exeter, UK), was diluted 1:1000 in coating buffer and the wells preincubated overnight at 4 °C. Blocking buffer (1% BSA-PBS) was added for 1 h followed by the load in duplicate of either serial 5-fold dilutions of recombinant NSE protein (0.008–5 μg/ml; Abcam) to generate a standard curve, or 2-fold dilutions of recombinant SYP protein (0.34–5.5 μg/ml; Abnova, Taipei City, Taiwan), homogenates at a 1:10 or blanks. Two hours later, peroxidase-labelled, mouse monoclonal anti-NSE (Abcam) or biotinylated anti-mouse IgG for SYP detection (Vector Laboratories), was added and incubated in the dark for 2 h.

#### Measurement of PSD95

Homogenate samples were diluted 1:20 and incubated in duplicate alongside blanks and a standard curve, comprising 3-fold dilutions of recombinant PSD95 protein (3.75–910.1 ng/ml; Abnova), for 2 h at 26 °C. Primary antibody (PSD95, clone 7E3-1B8, Sigma Aldrich, Gillingham, UK) diluted to 1:3000 was incubated for 2 h at 26 °C followed by the addition of a secondary antibody (HRP-labelled anti-mouse IgG; Vector Laboratories). The final stage of each ELISA involved the addition of a peroxidase substrate (R&D Systems, Minneapolis, USA).

For all of the ELISAs, absorbance was read at 450 nm in a multi-mode microplate reader (FLUOstar OPTIMA, BMG Labtech) and absolute protein levels (μg/ml) were determined by interpolation against the relevant standard curve.

### MesoScale discovery multiplex assay

Inflammatory proteins were measured on the V-Plex MSD electrochemiluminescence multi-spot assay platform (MesoScale Diagnostics, Rockville USA).100 mg of fresh frozen grey matter from AD cases (*n* = 67) was homogenised at a tissue concentration of 20% *w*/*v* in RIPA lysis buffer (Thermo Fisher Scientific) by use of a handheld homogeniser (Thermo Fisher Scientific); the buffer was supplemented with protease inhibitors (Complete Mini, Sigma Aldrich) and phosphatase inhibitors (Thermo Fisher Scientific). Total protein concentration in the supernatant was measured by BCA Protein Assay Kit (Thermo Fisher Scientific). 12.5μl of brain homogenate (1:4 dilution) was used for each assay according to the manufacturer’s protocol. The following V-PLEX human biomarker 40-PLEX kits were used: pro-inflammatory panel 1, cytokine panel 1 and vascular injury panel 2. Each plate was imaged on the Meso QuickplexSQ120 (MesoScale Discovery) according to manufacturers’ instructions for 384-well plates to obtain absolute protein levels (pg/ml). Frozen blocks from 4 controls and 2 multiple sclerosis brains containing chronic inactive, acute and chronic active lesions were used as negative and positive controls, respectively.

### qPCR

Inflammatory gene expression was determined by qPCR. mRNA was isolated in TRI-Reagent (Thermo Fisher Scientific) from 100 mg of fresh frozen grey matter from Alzheimer’s cases (*n* = 67). Reverse transcription (RT) was performed using the high capacity cDNA reverse transcription kit (Thermo Fisher Scientific). Gene expression was analysed using TaqMan gene expression assays (Thermo Fisher Scientific; Additional file 1: Table S2) and TaqMan universal PCR master mix in a 7900HT fast qPCR system machine (Thermo Fisher Scientific). RT and qPCR were performed as previously described [43, 52]. The same control and multiple sclerosis tissue as for the MSD protocol was utilized.

Data were extracted using SDS version 2.13 software (Thermo Fisher Scientific). The mRNA levels of the inflammatory markers were calibrated against GAPDH mRNA and the fold difference between groups was calculated by the 2^−ΔΔCt^ method.

### Statistical analysis

For all immunohistochemistry and assay data, the normality of distribution across each group was assessed by examination of quantile-quantile plots (not shown). *Immunohistochemistry:* The means within each group were compared by two-way ANOVA to assess the effect of Alzheimer’s disease or/and systemic infection on different proteins in the grey and white matter. Data were presented as mean ± standard deviation (SD). If an “Alzheimer’s disease” or “infection” effect was observed on its own, the contrast model was applied. If an interaction Alzheimer’s disease*infection was found, one-way ANOVA was performed to delineate interaction hierarchy. Correlations between the grey and white matter were assessed for each inflammatory marker; based on the normality of the data, Pearson’s (parametric) or Spearman’s (non-parametric) test was applied. For the CD3+ T cells, Fisher’s exact test was used for comparisons between subgroups with respect to the presence of the cells between group in the parenchyma or perivascular spaces in the grey or white matter. *ELISA, MSD assay and qPCR:* Mann-Whitney U-test was used for comparisons between AD- and AD+ groups. Data were presented as median with interquartile range (IQR). All analyses were performed with SPSS software (version 24, IBM). *P* values less than 0.05 for intergroup comparisons and 0.01 for correlations were considered statistically significant. Graphs were prepared with GraphPad Prism software (version 6, La Jolla, CA) and figures with Photoshop CS6 (version 13.0 × 64, Adobe).

## Results

### Neuropathology

To investigate whether systemic infection modifies key neuropathological features of AD, we performed immunohistochemistry to compare Aβ and ptau loads between the four groups, and ELISA to compare pre- and post-synaptic proteins in the two Alzheimer’s groups. Systemic infection did not change Aβ or ptau loads in either control or Alzheimer’s patients. However, as expected, AD was associated with increased Aβ (*p* < 0.001) and ptau (*p* < 0.001) compared to controls, irrespective of systemic infection (Table 2). Similarly, systemic infection did not affect the concentration of SYP or PSD95, or the ratio between these proteins, in AD (AD+ vs. AD-; Table 3).

**Table 2:** Quantification of the neuropathological changes. Amyloid (A)β and hyperphosphorylated (p)tau loads (%) in control and Alzheimer’s disease cases detected by immunohistochemistry

Protein load (%)	Ctrl-	Ctrl+	AD-	AD+	Mean difference (95% CI)	*P* value
Aβ load	2.66 ± 3.38	2.97 ± 3.91	7.49 ± 3.37	6.46 ± 2.95	4.15 (2.82, 5.48)	*< 0.001*
pTau	0.01 ± 0.20	0.04 ± 0.11	2.20 ± 3.52	2.02 ± 2.20	2.09 (1.18, 2.98)	*< 0.001*

Values are mean ± SD; *p* value by 2-way ANOVA test; significant *p* value in italic

*Ctrl* neurologically/cognitively normal controls, *AD* Alzheimer’s disease cases, − died without infection, + died with infection, *SD* standard deviation, *CI* confidence interval

**Table 3:** Quantification of the neuropathological changes. Synaptic proteins synaptophysin (SYP) and PSD-95 in Alzheimer’s disease cases revealed by ELISA (μg/ml)

Protein concentration (μg/ml)	AD-	AD+	*P* value
SYP	1.06 (0.71, 1.74)	1.39 (0.74, 2.46)	0.242
PSD-95	1.95 (0.10, 3.35)	1.92 (1.04, 2.48)	0.374
SYP/PSD-95	0.54 (0.34, 1.15)	0.76 (0.40, 1.50)	0.269

Values are median ± IQR; *p* value by Mann-Whitney test

*SYP* Synaptophysin, *AD* Alzheimer’s disease cases, − died without systemic infection, + died with systemic infection, *IQR* interquartile range

### Neuroinflammatory environment

To assess the effect of systemic infection on the neuroinflammatory environment in AD, we used the MSD platform to measure the levels of IFNγ, IL1β, IL2, IL4, IL6, IL8, IL10, IL12p70, IL13, TNFα, IL1α, IL5, IL7, IL12/IL23p40, IL15, IL16, IL17A, GM-CSF, TNFβ and VEGF in AD- and AD+ groups. Significant differences in AD+ cases were as follows: an increase in pro-inflammatory IL6 (1.5-fold, *p* = 0.047) and a decrease in cytokines IL5 (2.0-fold, *p* = 0.007), IL7 (2.6-fold, *p* = 0.002), IL12/IL23p40 (2.3-fold, *p* = 0.001), IL15 (1.6-fold, *p* = 0.008), IL16 (2.4-fold, *p* < 0.001) and IL17A (2.4-fold, *p* < 0.001) (Table 4).

**Table 4:** Comparison of inflammatory proteins in Alzheimer’s cases detected by V-PLEX Meso Scale Discovery Multiplex Assays

	AD-	AD+	*P* value	Fold change
Pro-inflammatory Panel 1 (pg/ml)				
IFN γ	0.18 (0.00, 0.87)	0.00 (0.00, 0.63)	0.266	
IL1 β	0.00 (0.00, 0.65)	0.36 (0.00, 0.95)	0.097	
IL2	0.32 (0.16, 0.56)	0.24 (0.00, 0.51)	0.393	
IL4	0.33 (0.27, 045)	0.33 (0.25, 0.40)	0.834	
IL6	2.74 (1.48, 4.35)	4.09 (2.14, 11.45)	*0.047*	1.5
IL8	15.35 (9.77, 31.44)	17.44 (11.46, 41.99)	0.242	
IL10	0.05 (0.00, 0.21)	0.08 (0.00, 0.20)	0.747	
IL12p70	1.60 (1.25, 2.21)	1.81 (1.17, 2.06)	0.736	
IL13	10.95 (9.50, 16.72)	11.70 (9.51, 15.29)	0.869	
TNFα	0.49 (0.37, 0.69)	0.57 (0.23, 0.72)	0.874	
Cytokines Panel 1 (pg/ml)				
IL1α	0.67 (0.00, 2.42)	0.46 (0.00, 2.64)	0.781	
IL5	0.08 (0.04, 0.18)	0.04 (0.01, 0.08)	*0.007*	−2.0
IL7	1.32 (0.81, 1.88)	0.54 (0.26, 1.09)	*0.002*	−2.6
IL12/IL23p40	0.70 (0.45, 1.14)	0.31 (0.13, 0.76)	*0.001*	− 2.3
IL15	6.32 (4.95, 8.24)	3.88 (2.42, 6.95)	*0.008*	−1.6
IL16	614.82 (404–13, 1031.83)	261.12 (151.69, 468.25)	*< 0.001*	−2.4
IL17A	4.57 (3.53, 5.04)	1.90 (1.05, 4.03)	*< 0.001*	−2.4
GM-CSF	0.70 (0.03, 0.14)	0.04 (0.00, 0.14)	0.463	
TNFβ	0.00 (0.00, 0.05)	0.00 (0.00, 0.02)	0.561	
VEGF	10.94 (4.78, 21.70)	7.75 (2.70, 17.52)	0.242	

Values are median with IQR; *p* value by Mann-Whitney test; significant *p* values in italic

Fold change, AD+ vs. AD-

*AD* Alzheimer’s disease cases, − died without systemic infection, + died with systemic infection, *IQR* interquartile range

**Fig. 1:**
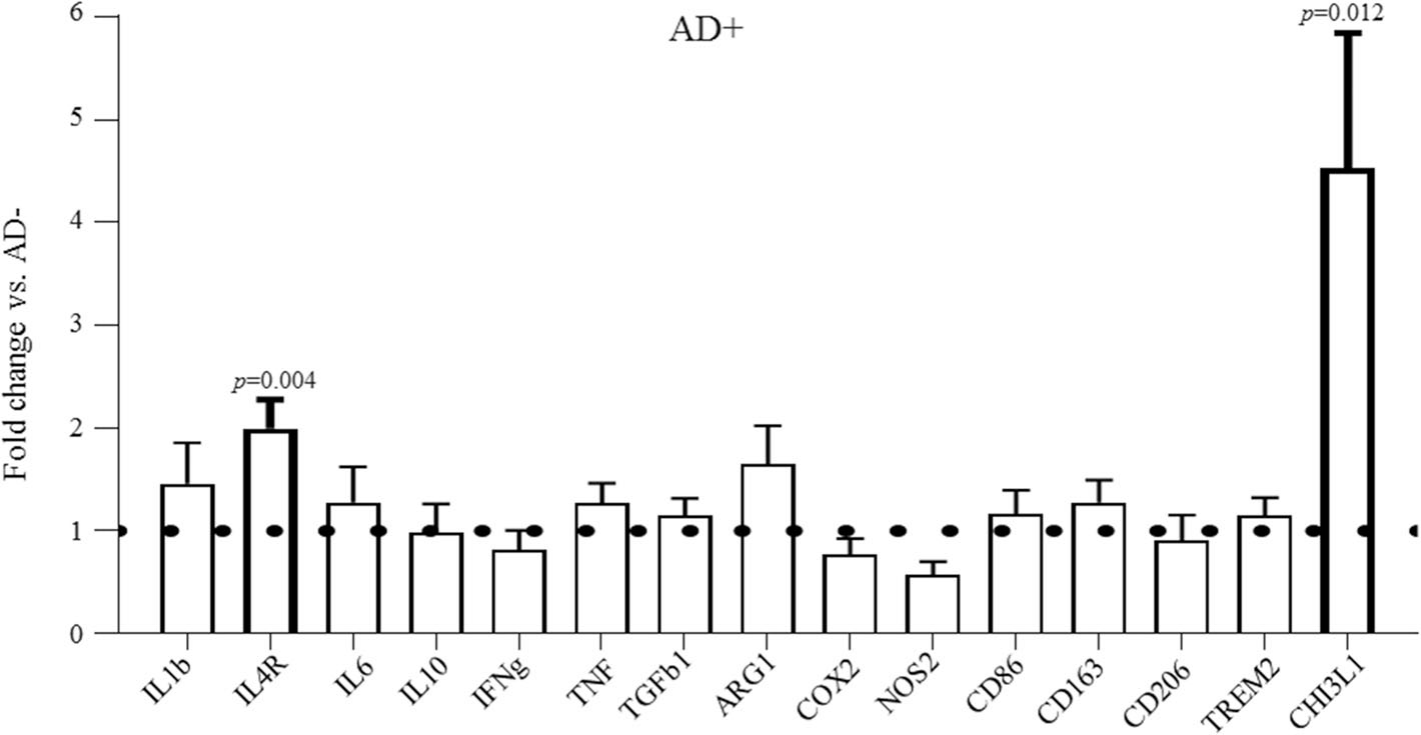
Expression of inflammatory molecules in the presence of systemic infection in Alzheimer’s disease using quantitative real-time PCR. The levels of indicated transcripts are normalised to *GAPDH*, and the mRNA Alzheimer’s disease without systemic infection (AD-) levels are arbitrary set as 1. The bar graph shows the fold difference in mRNA of inflammatory markers and indicates significant increased anti-inflammatory gene transcripts *CHI3L1* (*p* = 0.012) and *IL4R* (*p* = 0.04) in Alzheimer’s disease with (AD+) compared to without systemic infection (AD-)

To investigate the role of systemic infection further in Alzheimer’s cases, we used TaqMan qPCR to compare the fold difference in mRNA levels between AD+ and AD- cases for cytokines and cytokine receptors (*IL1b, IL4R, IL6, IL10, IFNg, TNF, TGFb1*), enzymes (*ARG1, COX2, NOS2*), receptors (*CD86, CD163, CD206, TREM2,*) and the anti-inflammatory marker *CHI3L1 (*Chitinase 3-Like 1), relative to *GAPDH* mRNA. An increase in *IL4R* (2-fold, *p* = 0.004) and *CHI3L1* (2.2-fold, *p* = 0.012) mRNA was detected in AD+ compared to AD- (Fig. 1).

### Microglia

Several markers associated with specific microglial functions were investigated by immunohistochemistry in grey and white matter. These included: Iba1, a marker of microglial motility [50, 51]; CD68, a lysosomal/endosomal-associated transmembrane glycoprotein associated with phagocytosis [44, 57]; HLA-DR, necessary for antigen-presentation and involved in the non-self-recognition [44, 63]; and CCR2, a microglial chemokine receptor involved in mononuclear phagocyte infiltration in mouse brain [21, 22]. FcγRs, as central effectors of immunoglobulin (Ig)G-mediated immune responses [48] were examined using CD64 (FcγRI), a high-affinity activating receptor [67]; CD32a (FcγRIIa) and CD16 (FcγRIII), both low-affinity activating receptors [48]; and CD32b (FcγRIIb) a low affinity inhibitory receptor [28]. In view of the qPCR findings, we also examined the anti-inflammatory proteins CHI3L1 and IL4R [8, 12].

**Fig. 2:**
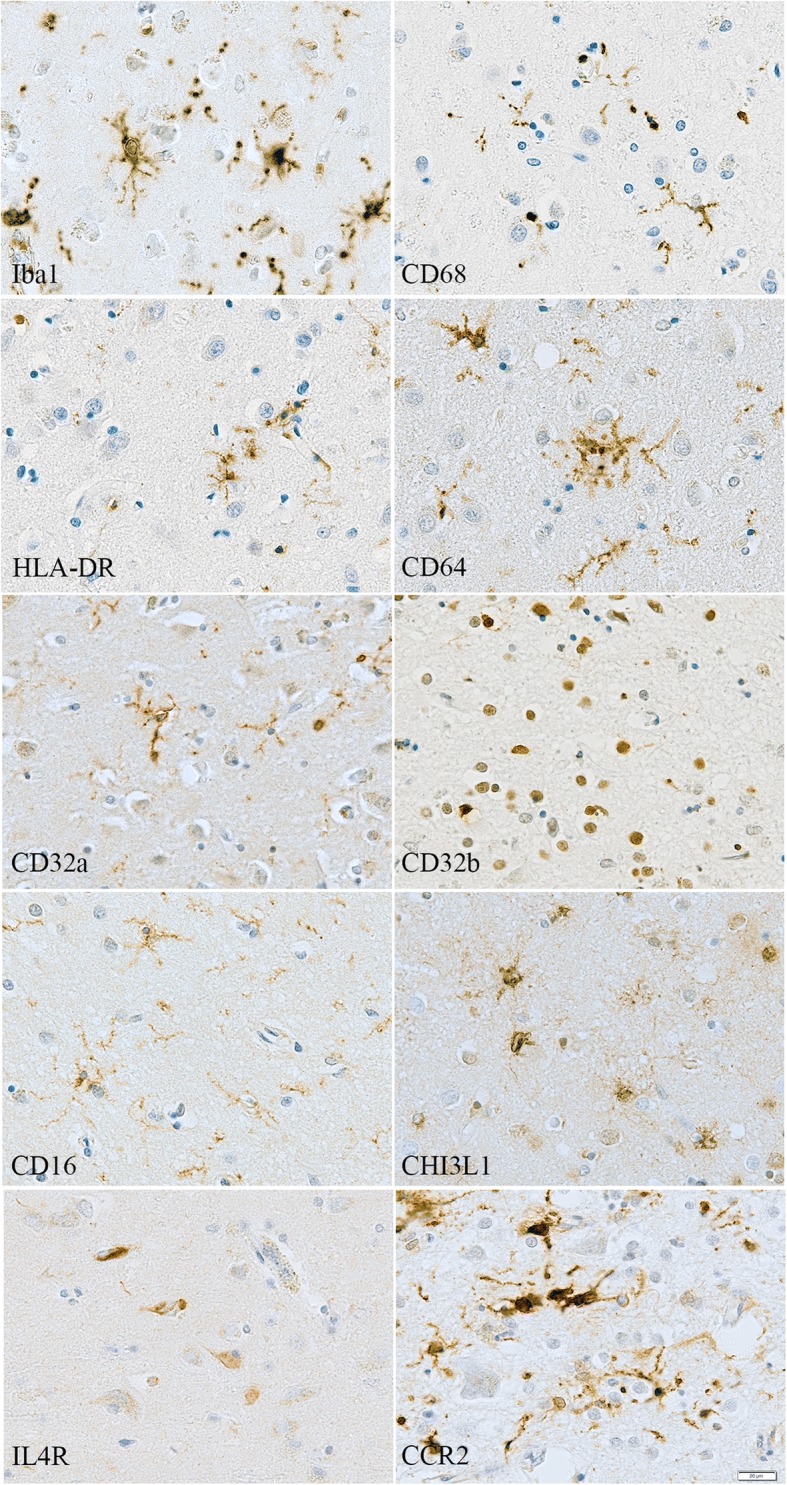
Illustration of the immunostaining obtained with the different inflammatory markers in Alzheimer’s disease. Counterstaining: Haematoxylin, Scale bar = 20 μm

Immunohistochemistry showed the following main cell-types expressing these proteins: antibodies to Iba1, CD68, HLA-DR, CD64 and CD16 immunolabelled microglia and perivascular macrophages; CD32a was also present in some neurons; CHI3L1 was detectable mainly in microglia as well as CCR2, as expected. CD32b, the only inhibitory FcγR, and IL4R were expressed in neurons, with IL4R antibody labelling tangles and neuropil threads in the Alzheimer’s cases (Fig. 2, Additional file 1: Table S1).

Quantification of the immunolabelling (Table 5) in the grey matter indicated that: (i) CD68 (*p* = 0.026), CD64 (*p* = 0.002), CHI3L1 (*p* = 0.016), IL4R (*p* = 0.005) and CCR2 (*p* = 0.010) loads were increased in AD irrespective of systemic infection; and (ii) CD16 load was affected by both AD and systemic infection (*p* = 0.027) such that CD16 expression was lower in AD with systemic infection (AD+) compared to AD without systemic infection (AD-). In the white matter, CD32a was decreased by AD (*p* = 0.030) independent of systemic infection. Both CD68 (*p* = 0.015) and CD64 (*p* = 0.017) were affected by systemic infection in AD, with decreased CD68 and increased CD64 loads in AD+ vs. AD-. The other inflammatory markers were not modified by either AD or systemic infection.

**Table 5:** Comparison of the inflammatory protein loads (%) in control and Alzheimer’s cases

	Ctrl-	Ctrl+	AD-	AD+	Mean difference (95% CI)	P value
Grey Matter						
Iba1	1.63 ± 0.88	1.81 ± 1.14	1.31 ± 0.75	1.35 ± 1.14		ns
CD68^a^	0.21 ± 0.08	0.25 ± 0.05	0.29 ± 0.14	0.28 ± 0.14	0.06 (0.007, 0.103)	*0.026*
HLA-DR	0.03 ± 0.05	0.04 ± 0.07	0.12 ± 0.16	0.11 ± 0.27		ns
CD64^a^	2.01 ± 0.85	2.26 ± 1.35	3.16 ± 1.57	2.97 ± 1.55	0.93 (0.36, 1.45)	*0.002*
CD32a	0.43 ± 0.33	0.46 ± 0.61	0.45 ± 0.58	0.36 ± 0.46		ns
CD32b^a^	0.08 ± 0.10	0.10 ± 0.10	0.29 ± 0.52	0.42 ± 1.00	0.27 (−0.001, 0.54)	ns
CD16^b^	0.35 ± 0.39	0.86 ± 1.25	0.98 ± 0.90	0.67 ± 0.93	Ctrl+: 0.51 (− 0.07, 1.08)	0.084
					AD-: 0.62 (0.13, 1.12)	*0.014*
					AD+: 0.31 (−0.15, 0.78)	0.179
CHI3L1^a^	0.24 ± 0.22	0.37 ± 0.36	0.57 ± 0.62	0.73 ± 0.96	0.34 (0.07, 0.62)	*0.016*
IL4R^a^	0.09 ± 0.08	0.07 ± 0.06	0.23 ± 0.33	0.20 ± 0.24	0.14 (0.04, 0.23)	*0.005*
CCR2^a^	0.12 ± 0.14	0.09 ± 0.08	0.74 ± 1.49	0.38 ± 0.50	0.46 (0.11, 0.80)	*0.010*
White Matter						
Iba1	1.46 ± 1.03	2.13 ± 1.52	1.27 ± 1.02	1.43 ± 1.13		ns
CD68^b^	0.11 ± 0.10	0.23 ± 0.17	0.36 ± 0.22	0.28 ± 0.24	Ctrl+: 0.12 (− 0.01, 0.25)	0.076
					AD-: 0.25 (0.14, 037)	*< 0.001*
					AD+: 0.17 (0.06, 0.27)	*0.003*
HLA-DR	0.05 ± 0.08	0.03 ± 0.06	0.15 ± 0.24	0.09 ± 0.23		ns
CD64^b^	0.70 ± 0.42	1.95 ± 1.46	1.36 ± 0.89	1.65 ± 1.02	Ctrl+: 1.25 (0.63, 1.87)	*< 0.001*
					AD-: 0.66 (0.12, 1.2)	*0.017*
					AD+: 0.95 (0.45, 1.45)	*< 0.001*
CD32a^a^	0.58 ± 0.67	0.48 ± 0.52	0.34 ± 0.46	0.27 ± 0.37	−0.23 (− 0.43, 0.02)	*0.030*
CD16	0.10 ± 0.14	0.31 ± 0.51	0.28 ± 0.30	0.23 ± 0.49		ns
CHI3L1	0.23 ± 0.26	0.50 ± 0.57	0.69 ± 0.73	0.62 ± 1.12		ns
CCR2	0.06 ± 0.11	0.12 ± 0.13	0.30 ± 0.55	0.18 ± 0.46		ns

Values are mean ± SD; significant *p* value in italic

^a^Alzheimer’s effect

^b^One-way ANOVA test performed following significant Alzheimer’s disease*infection interaction on the 2-way ANOVA analysis

ns, non-significant following the 2-way ANOVA analysis

*Ctrl* neurologically/cognitively normal controls, *AD* Alzheimer’s disease cases, − died without systemic infection, + died with systemic infection, *SD* standard deviation, *CI* confidence interval

We then explored the possible relationship between grey and white matter neuroinflammatory markers in the different subgroups to assess whether some of the markers were associated with the presence of systemic infection (Table 6). We found a grey-white matter correlation for HLA-DR, CD32b, CD16 and CHI3L1 regardless of subgroup. Grey-white matter correlation for Iba1 was found only in controls (Ctrl- and Ctrl+); grey-white matter correlation for CD68 and CCR2 was limited to brains affected by AD (AD- and AD+). Interestingly, the grey-white matter correlation for CD64 was restricted to brains from donors with systemic infection. (i.e. present in both Ctrl+ and AD+).

**Table 6:** Correlations of neuroinflammation-related markers between the grey and the white matter in control and Alzheimer’s cases

Grey vs white matter	Iba1	CD68	HLA-DR	CD64	CD32a	CD16	CHI3L1	CCR2
Ctrl-	ρ = 0.641***	ns	ρ = 0.731***	ns	ρ = 0.666***	ρ = 0.893***	ρ = 0.849***	ns
Ctrl+	*r* = 0.724**	ns	ρ = 0.766***	*r* = 0.763***	ρ = 0.707**	ρ = 0.903***	ρ = 0.768***	ns
AD-	ns	ρ = 0.699***	ρ = 0.917***	ns	ρ = 0.956***	ρ = 0.842***	ρ = 0.821***	ρ = 0.892***
AD+	ns	ρ = 0.771***	ρ = 0.925***	*r* = 0.620***	ρ = 0.866***	ρ = 0.801***	ρ = 0.896***	ρ = 0.851***

ρ, Spearman; r, Pearson; **p ≤ 0.01; ***p ≤ 0.001

*Ctrl* neurologically/cognitively normal controls, *AD* Alzheimer’s disease cases, − died without systemic infection, + died with systemic infection

### T lymphocytes

**Fig. 3:**
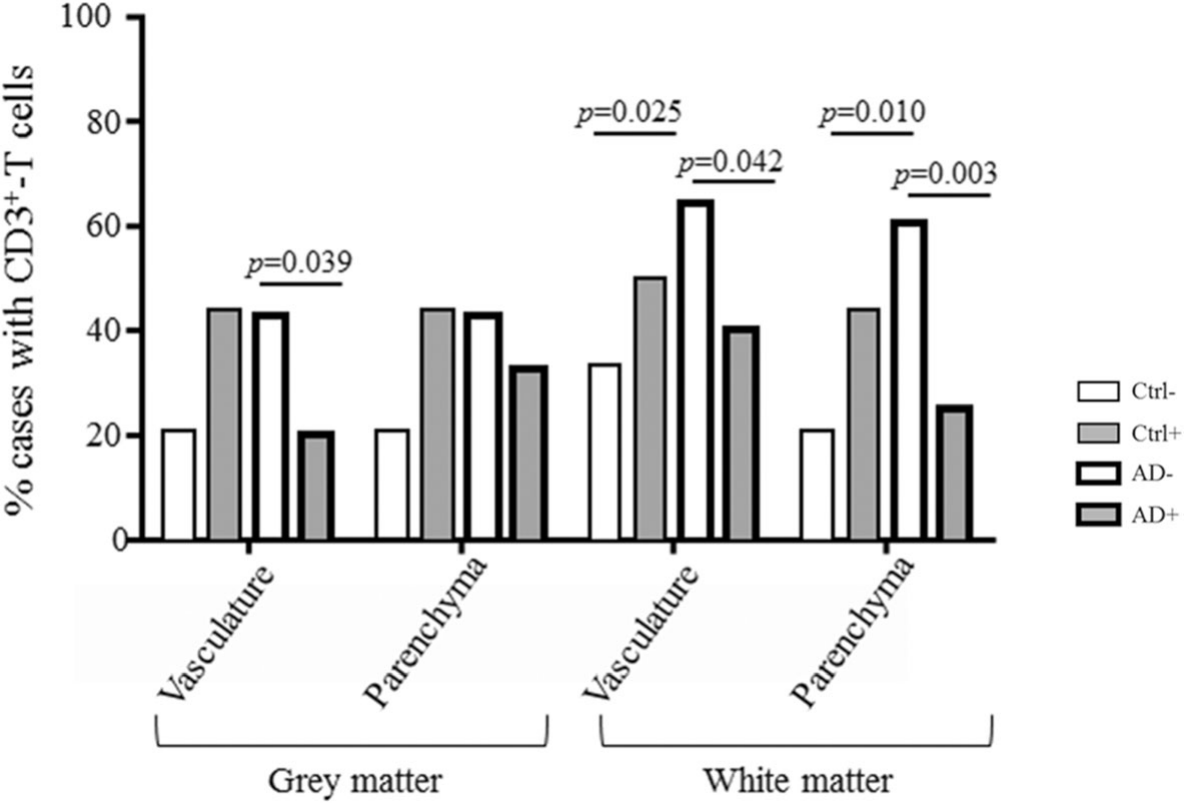
Quantification of the CD3-positive T cells as percentage of cases presenting T cells in the blood vessels and/or the parenchyma in the grey and white matter, in the controls and Alzheimer’s cases in the presence or absence of systemic infection at the time of death. The effect of Alzheimer’s disease was detected in the white matter with increased T cells in the blood vessels (*p* = 0.025) and parenchyma (*p* = 0.010). An effect of infection was observed in Alzheimer’s disease with fewer T cells in the Alzheimer’s disease with systemic infection group in the grey matter blood vessels (*p* = 0.039), and the white matter (blood vessels: *p* = 0.042; parenchyma: *p* = 0.003)

We used immunohistochemistry for the pan-T cell marker CD3 [9] to investigate the relationship between systemic infection and T cell recruitment into the perivascular compartment and brain parenchyma in the grey and white matter. Systemic infection influenced T cells recruitment, with fewer cases displaying T cells in AD+ vs. AD- (grey matter: blood vessels, *p* = 0.039; white matter: blood vessels, *p* = 0.042; parenchyma, *p* = 0.003). In the absence of systemic infection, we confirm the presence of sparse T cells in AD brain [59] (Fig. 3).

### Vascular damage

To investigate whether the neuroinflammatory changes after systemic infection might reflect vascular damage, we used the MSD platform to compare the levels of CRP, ICAM1, SAA, and VCAM1 between AD+ and AD- brains. No significant differences were observed (Additional file 1: Table S3).

## Discussion

Our aim was to examine whether terminal systemic infection modified AD pathology, synaptic proteins and neuroinflammation. We found that systemic infection was associated with downregulation of a range of pro-inflammatory markers and reduced T cell recruitment in the brain, but had no effect on Aβ, ptau, or synaptic proteins. In addition, systemic infection was associated with upregulated expression of the anti-inflammatory genes *IL4R* and *CHI3L1*, in keeping with an immunosuppressive environment [11, 24].

Our study has limitations. Firstly, this was a retrospective observational study rather than a prospective experimental study. As an end-stage study, it was not possible to explore the temporal relationship between the different markers investigated, and thus the analysis was limited to assessment of the late-stage consequences of AD and systemic infection present at the time of death. Case selection with respect to the presence or absence of terminal infection relied on *post-mortem* findings and death certificates, and it is possible that the groups without systemic infection may have included some individuals with early, unrecognised infections. Conversely, in the groups with systemic infection, the infective process may have been too acute (i.e. short-lived) to have had major effects on brain inflammation. In addition, the lack of cytokine and protein measures in the control groups meant we could not provide information on the environment induced by systemic infection in the absence of Alzheimer’s disease. Nevertheless, to our knowledge, this is the first neuropathological study of the effects of systemic infection on the neuroinflammatory environment and disease response in human AD. The major advantage of studying the human brain in this way is that it is a study of the disease itself rather than an experimental model of the disease. The novelty of our study resides in the combined quantitative assessment of multiple microglial markers with known functions, the neuroinflammatory environment and the neuropathological features of AD.

### The neuroinflammatory environment in systemic infection

In AD, systemic infection was associated with increased IL6 and decreased levels of several pro-inflammatory cytokines. IL6 has been extensively studied in AD, in which there are elevated levels in the blood and brain [39], associated with cognitive decline [35]. In the context of systemic infection in AD, raised serum IL6 was related to increased neuropsychiatric symptoms characteristic of sickness behaviour [29], consistent with our observation of a 1.5-fold elevation in IL6 in the brain in the Alzheimer’s cases with systemic infection.

Systemic infection in AD was also associated with a reduction in several pro-inflammatory cytokines, mainly associated with the adaptive immune system. The few studies that have examined their role in AD have found: (i) elevated serum IL7 in early to mild AD [20]; (ii) elevated IL12p40 levels in the cerebrospinal fluid (CSF) of Alzheimer’s patients [68]; (iii) administration of IL12p40 subunit blocker enhanced microglial phagocytosis and reduced inflammation in Aβ transgenic mice [68]; (iv) raised IL15 levels in the CSF and serum of Alzheimer’s patients correlated with severity of cognitive dysfunction [6, 58]; (v) increased peripheral IL16 in AD [19]; and overexpression of IL17A decreased soluble Aβ levels without exacerbating neuroinflammation in a mouse model of Aβ accumulation [73].

Our observed decrease in expression by more than 50% of several pro-inflammatory proteins with systemic infection should be considered in relation to the upregulation of the anti-inflammatory genes *IL4R* and *CHI3L1*. The role of IL4 in AD is uncertain: higher peripheral IL4 was found in mild cognitive impairment patients but not in dementia; increased disease severity was associated with lower levels of IL4 [36]. These findings may reflect a role for inflammation early in the disease process, consistent with genetic studies [33]. The significance of our observation of increased IL4R in AD with a similar immunolabelling pattern to that of ptau in AD brains is unclear. Loss of the normal immunoregulatory interaction between microglia and neurons, possibly through loss of the microglial regulatory protein CD200 [69], could lead microglia towards an anti-inflammatory profile, as suggested by previous studies [13, 34]. CHI3LI downregulates the cellular responses to pro-inflammatory cytokines TNFα and IL1β in vitro [40], implying an important role in regulating the inflammatory processes [37]. In AD, CHI3L1 was reported upregulated in the brain [13], and detected in the CSF of patients [72], and has been suggested as a biomarker for preclinical [14] and early AD [10, 26]. Interestingly, CHI3L1 raised levels were associated with markers of neurodegeneration in the preclinical stages of AD [2] and more specifically with tau-related neurodegeneration [3], perhaps related to IL4 expression. Indeed, BV2 mouse microglia treated with IL4 and IL13 upregulated the alternative activation genes [13], consistent with an association between IL4/IL4R and CHI3L1. IL4R and CHI3L1 seem usually to be expressed together and associated with an immunosuppressive environment.

### Microglia and T cells in systemic infection

Systemic infection in Alzheimer’s disease was associated with decreased CD68, CD16 (FcγRIII) and increased CD64 (FcγRI) proteins. The activating and inhibitory FcγRs, together generate a balanced immune response, associated with the production of a mixture of pro- and anti-inflammatory mediators [42, 46] and increased phagocytic activity [70]. FcγR expression was observed on microglia in normal and Alzheimer’s human brain [53]; however, that study did not distinguish between the activating and inhibitory receptors. Modulation of microglial FcγRs was reported after acute systemic infection in chronic neurodegenerative disease in rodents: prion-infected mice challenged with a single intra-peritoneal lipopolysaccharide (LPS) injection upregulated FcγRIII and FcγRIV, but not other microglial receptors including the inhibitory FcγRII [41]. Our data are consistent of an effect of systemic infection on FcγRs in AD and support a role for these receptors in the disease pathogenesis, but decrease in FcγRs in AD with systemic infection group again emphasizes the difference between the human disease and experimental models of neuroinflammation. The decreased CD16 and CD68, reflecting reduced phagocytic activity, is consistent with an immunosuppressive environment that might incapacitate the immune system so that it cannot respond appropriately to the disease. The increase in CD64, the FcγR with the highest affinity for IgG, in the white matter in the presence of systemic infection, may reflect the presence of more susceptible/primed or less immunosuppressive microglia in the white than in the grey matter, perhaps due the absence of pathology in the white matter, or differences in the blood-brain barrier.

The number of T Cells in AD [64, 74] is diminished in the presence of systemic infection, as would be expected in the context of an immunosuppressive environment and with a dynamic communication between the systemic and brain immune systems. Measurement of CRP in serum is used clinically as a marker of systemic inflammatory processes but blood samples were not available for our cases. We performed the CRP measurement in brain tissue, but of note, the presence of systemic infection in the AD subjects was not reflected in. It is acknowledged that the cardinal signs of infection in the elderly may be absent or blunted in 20–30% of patients [1, 49]. In the elderly, serum CRP begins to rise 6 h after a bacterial infection with the peak reached after 48 h and a half-life of 19 h [5]. Our CRP finding may be due to (i) absence of a rise in serum CRP in our patients, (ii) a dilution effect resulting from the much lower concentration of the protein in brain than serum or (iii) an inadequate survival time for a CRP response to have developed.

Interestingly, associations between microglial markers in the grey and white matter highlighted (i) Iba1 associated with control groups independently of systemic infection, maybe reflecting microglial motility, a function essential to healthy brain [44, 47]; (ii) CD68 (phagocytosis) and CCR2 (monocyte recruitment) as markers of neurodegeneration in AD independently of systemic infection; and (iii) CD64 as a potential marker of systemic infection whatever the disease status [44].

### The neuroinflammatory environment in AD

We observed increased anti-inflammatory CHI3L1, IL4R, CD64 and CD32b proteins, potentially highlighting an anti-inflammatory maybe immunosuppressive environment in AD independent of systemic infection. Another study reported upregulation of alternative activation genes in experimental models and AD brains [13], and we previously showed in the Cognitive Function in Ageing (CFAS) cohort that CD64 was associated with dementia [44]. AD was also associated with increased phagocytosis (CD68), monocyte recruitment (CCR2), and immune responses mediated by CD64 receptor, mainly in the grey matter, in keeping with the distribution of Alzheimer’s pathology and as previously reported in human and experimental studies [7, 23, 27, 32, 44].

### Systemic infection and Alzheimer’s neuropathology

Systemic infection did not affect Aβ, ptau or synaptic protein levels. This could be explained by (i) a saturation effect with the proteins having reached a plateau at late-stage disease [60]; (ii) a short interval between the onset of systemic infection and death not allowing time for the infection to modify protein levels via an altered neuroinflammatory environment; (iii) the immunosuppressive environment already present in AD and enhanced with systemic infection; or (iv) the absence of a relationship between the two events and these proteins.

## Conclusion

In conclusion, our study suggests that end-stage AD is associated with an anti-inflammatory (i.e. reducing or counteracting inflammation) brain environment, potentially immunosuppressive in the context of systemic infection. This underlines the difference between human disease and experimental models, that latter suggesting that a pro-inflammatory environment with enhanced neuronal loss is driven by systemic infection, and the assumption that sickness behaviour associated with raised peripheral TNFα and accelerated cognitive decline is due to an enhanced cerebral inflammation. Factors that could contribute to the difference in immune responses include the specific-pathogen-free environment in which the experimental animals are bred (unlike the human patients, who have been subjected to a lifetime of infections), and the experimental design. Indeed, a recent study in mice demonstrated that repeated peripheral LPS injections modified microglia and induced immune tolerance within the brain [71]. Based on the current knowledge, we suggest that early in the development of AD, microglia primed by systemic infection respond to the disease in a detrimental manner (i.e. causing sickness behaviour, neuronal loss, increased pathology), but that over time, repeated systemic infections may induce an immunosuppressive environment within the brain so that towards the end-stage of AD, there is marked downregulation of microglial inflammation, with equally deleterious consequences as evidenced by the accelerated cognitive decline [30].

## Additional file

Additional file 1: **Table S1.** Characteristics of the primary antibodies, immunohistochemistry conditions and expression of the immunolabelling. **Table S2.** Primers and probes used for TaqMan qPCR (human sequences). **Table S3.** Comparison of vascular proteins in Alzheimer’s cases detected by V-PLEX Meso Scale Discovery Multiplex Assays. (PDF 51 kb)

## Abbreviations

ARG: Arginine
Aβ: Amyloid-β
CCR: CC chemokine receptor
CHI3L1: Chitinase 3-Like 1
CNS: Central nervous system
COX: Cyclooxygenase
CRP: C reactive protein
CSF: Cerebrospinal fluid
ELISA: Enzyme-linked immunosorbent assay
GAPDH: Glyceraldehyde 3-phosphate dehydrogenase
GM-CSF: Granulocyte macrophage colony-stimulating factor
HLA: Human leukocyte complex
HRP: Horseradish peroxidase
Iba1: Ionized calcium-binding adaptor molecule 1
ICAM: Intercellular adhesion molecule
IFN: Interferon
IL: Interleukin
LPS: Lipopolysaccharide
MCI: Mild cognitive impairment
MMSE: Mini-mental state examination
MSD: MesoScale Discovery
NOS: Nitrous oxide system
NSE: Neuron specific enolase
PET: Positron emission tomography
PSD: Postsynaptic density
ptau: Hyperphosphorylated tau
SAA: Serum amyloid A
SYP: Synaptophysin
TGF: Transforming growth factor
TNF: Tumour necrosis factor
TREM: Triggering receptor expressed on myeloid cells
TSPO: 18-kDA translocator protein
VCAM: Vascular cell adhesion protein
VEGF: Vascular endothelial growth factor
