# Supplementary material for: Systemic infection modifies the neuroinflammatory response in late stage Alzheimer’s disease
Source: Acta Neuropathol Commun. 2018 Sep 7;6:88. doi: 10.1186/s40478-018-0592-3 (PMC6127939; doi:10.1186/s40478-018-0592-3)
Supplement: Supplementary file 1 — Table S1. Characteristics of the primary antibodies, immunohistochemistry conditions and expression of the immunolabelling. Table S2. Primers and probes used for TaqMan qPCR (human sequences). Table S3. Comparison of vascular proteins in Alzheimer’s cases detected by V-PLEX Meso Scale Discovery Multiplex Assays. (PDF 51 kb) [file 40478_2018_592_MOESM1_ESM.pdf]

Supplementary Table 1 – Characteristics of the primary antibodies, immunohistochemistry conditions and expression of the staining

Antigen (human)	Company	Host	Pre-treatment	Dilution	Predominant neuropathological features / cell type stained	Immune functions
**Amyloid (A)β**	Covance	Mouse, clone 4G8	80% formic acid and citrate buffer	1/2000	Diffuse and neuritic plaques, cerebral amyloid angiopathy	
**Phosphorylated Tau (pS202+pT205)**	Fisher Scientific	Mouse, clone AT8	Citrate buffer	1/500	Tangles, dystrophic neurites, neuropil threads	
**Iba1**	Wako	Rabbit	Citrate buffer	1/1000	Microglia, perivascular macrophages	Cytoplasmic protein involved in cytoskeletal reorganization, membrane ruffling of the microglial processes and actin cross-linking needed for cell migration [51], reflecting microglial motility and migration properties. Higher expression of Iba1 is associated with good MMSE and absence of dementia [44].
**CD68**	Abcam	Mouse (clone PG-M1)	0.5% pronase	1/50	Microglia, perivascular macrophages	CD68 labels lysosomal and endosomal transmembrane glycoprotein of microglia, indicating phagocytic activity [57]. CD68 is associated with worse MMSE, dementia and ptau [44].
**HLA-DP,DQ,DR**	Dako	Mouse, clone CR3/43	Citrate buffer	1/200	Microglia, perivascular macrophages	HLA-DR is a Major Histocompatibility Class (MHC) II cell surface receptor which presents antigens to cells of the immune system eliciting an immune response, involved in the non-self recognition and upregulated in inflammation [63]. HLA was identified as a genetic risk factor for Alzheimer’s disease [33].
**CD64 (FcgRI)**	R&D Systems	Goat	EDTA buffer	1/100	Microglia, perivascular macrophages	

**CD32a (FcgRIIa)**	Abcam	Mouse (clone 13D7)	EDTA buffer	1/2000	Microglia, perivascular macrophages and in some neurons	Fcγ receptors are central effectors of immunoglobulins (IgG) mediated immune response [48]. CD64 has high affinity for the Fc portion of IgG, triggering a monocyte/macrophage response [67]. CD64 expression reflects the presence of IgG in the brain and thus the involvement of systemic immunity [41]. CD32a/b and CD16 are low/medium affinity receptors for immune complex [48]. CD32b, the only inhibitory receptor, is usually co-expressed with activating FcγR on the majority of innate immune cells [8, 28].
**CD32b (FcgRIIb)**	Abcam	Rabbit (clone EP888Y)	EDTA buffer	1/4000	Neurons	
**CD16 (FcgRIII)**	R&D Systems	Goat	EDTA buffer	1/150	Microglia, perivascular macrophages, some monocytes and neurons	
**CHI3L1**	R&D Systems	Goat	Citrate buffer, PC	1/100	Microglia, some neurons	CHI3L1 (or YKL40), a highly evolutionary conserved secreted protein contains the conserved chitinase-like enzyme domain but lacks chitinolytic enzymatic activity. CHI3l1 is expressed by various cells [37], implicated in inflammatory disorders involving macrophage activation shown to be involved in promoting inflammation via regulation of M2/Th2-like immune reactions, leading to an immunosuppressive environment [11]. It is a potential neuroinflammatory biomarker for preclinical Alzheimer's disease [26].
**IL4R**	Sigma	Rabbit	EDTA buffer	1/100	Occasional healthy neurons in Ctrl, tangles and neuropil threads in Alzheimer's disease	IL4/IL4R are anti-inflammatory molecules participating to the alternative activation of macrophages [8, 24].
**CCR2**	Abcam	Rabbit, clone	EDTA buffer	1/500	Microglia, perivascular macrophages, monocytes	CC-chemokine receptor 2 is required for mononuclear phagocyte recruitment [22] and upregulated in Alzheimer's disease brains [32], but not expressed by microglia in mouse brain [23].
**CD3**	Dako	Mouse (clone F7.2.38)	None	1/200	T lymphocytes	CD3 is a T-cell co-receptor necessary to activate both $CD8^+$ and $CD4^+$ T cells [9], and thus considered as a pan-T cell marker.

Supplementary Table 2 - Primers and probes used for TaqMan qPCR (human sequences)

	Gene	Accession No.	Assay/Probe ID (Thermo Fisher Scientific)	Amplicon Length (bp)
**Cytokines/cytokine receptors**	***IL1β***	NM_000576.2	Hs01555410_m1	91
	IL6	NM_000600.4	Hs00985639_m1	66
	TNF	NM_000594.3	Hs99999043_m1	85
	IFN γ	NM_000619.2	Hs00989291_m1	73
	TGF β	NM_000660.5	Hs00998133_m1	57
	IL10	NM_000572.2	Hs00961622_m1	74
	IL4R	NM_000418.3, NM_001257406.1, NM_001257407.1, NM_001257997.1	Hs00166237_m1	70
**Enzymes**	***PTGS2 (COX2)***	NM_000963.3	Hs00153133_m1	75
	NOS2	NM_000625.4	Hs01075529_m1	67
	ARG1	NM_000045.3, NM_001244438.1	Hs00968979_m1	78
**Receptors**	***CD206 (MRC1)***	NM_002438.3	Hs00267207_m1	82
	CD86	NM_001206924.1, NM_001206925.1, NM_006889.4, NM_175862.4, NM_176892.1	Hs01567026_m1	104
	TREM2	NM_001271821.1, NM_018965.3	Hs00219132_m1	73
	CD163	NM_004244.5, NM_203416.3	Hs00174705_m1	72
**Others**	***CHI3L1***	NM_001276.2	Hs00609691_m1	77
	GAPDH	NM_001256799.2, NM_001289745.1, NM_001289746.1, NM_002046.5	Hs02758991_g1	157

Supplementary Table 3 - Comparison of vascular proteins in Alzheimer's cases detected by V-PLEX Meso Scale Discovery Multiplex Assays

Vascular injury panel 2 (pg/ml)	AD-	AD+	P value
CRP	53626.44 (13019.64, 85228.62)	55061.38 (27578.42, 90583.96)	0.461
ICAM-1	65379.43 (41726.25, 89186.55)	60035.26 (43099.03, 96183.34)	0.722
SAA	199894.72 (73367.75, 509940.34)	397225.57 (102854.00, 549630.35)	0.409
VCAM-1	3458.52 (3038.75, 4607.98)	3081.83 (2396.07, 4348.85)	0.158

Values are median with IQR; p value by Mann-Whitney test.
AD, Alzheimer's disease cases; -, without systemic infection; +, with systemic infection; IQR, interquartile range
